# Supplementary material for: Rapid turnover of life-cycle-related genes in the brown algae
Source: Genome Biol. 2019 Feb 14;20:35. doi: 10.1186/s13059-019-1630-6 (PMC6374913; doi:10.1186/s13059-019-1630-6)
Supplement: Supplementary file 3 — Protein alignments, corresponding Gblocks html file and CDS sequences for the lineage conserved gametophyte-biased genes. (ZIP 2109 kb) [file 13059_2019_1630_MOESM3_ESM.zip › Con_GA_alignments/Ec-18_003850.aa_ali.fasta-gb.htm]

Ec-18\_003850.aa.fasta.trans.aa\_ali.fasta


## Gblocks 0.91b Results

Processed file: **Ec-18\_003850.aa.fasta.trans.aa\_ali.fasta**  
Number of sequences: **2**  
Alignment assumed to be: **Protein**  
New number of positions: **254** (selected positions are underlined in blue)

```
                         10        20        30        40        50        60
                 =========+=========+=========+=========+=========+=========+
Ec-18_003850     MFIILRGLRPGTGSMPRCHIAEPDRLPGGRQSCDLMAIRTAAPNSNDVEERSRRSQDEGA
MSTRG.48448.1    ------------------------------------------------------------
                                                                             


                         70        80        90       100       110       120
                 =========+=========+=========+=========+=========+=========+
Ec-18_003850     GPRETSQKMLCTKIRNQAKELAALTERLQEEAAYSRLVEKRLVELDPEHSLPVTPHHLGR
MSTRG.48448.1    ------------------------------------------------------------
                                                                             


                        130       140       150       160       170       180
                 =========+=========+=========+=========+=========+=========+
Ec-18_003850     RSPESTIAHRSSGSVGGLGRGYHLQKADQLSNEEHDHDDGLRQGYEAAQARLRDAAQLIR
MSTRG.48448.1    ------------------------------------------------------------
                                                                             


                        190       200       210       220       230       240
                 =========+=========+=========+=========+=========+=========+
Ec-18_003850     TLREALESRDADANAAANRARKYQREADDLRRRLETSGGNVSRASKGGSGGGRVGENQTS
MSTRG.48448.1    ------------------------------------------------------------
                                                                             


                        250       260       270       280       290       300
                 =========+=========+=========+=========+=========+=========+
Ec-18_003850     SSPLGGEQGHGRELGIRFVDDDRARQHQQHVELSKDVERLEQKLREQREATTAARTEANG
MSTRG.48448.1    ------------------------------------------------------------
                                                                             


                        310       320       330       340       350       360
                 =========+=========+=========+=========+=========+=========+
Ec-18_003850     LHETLKQLQQSHHVGNLSSQREGAADDAKANAAELARQLARARSTAESSREENKNLRLQL
MSTRG.48448.1    ------------------------------------------------------------
                                                                             


                        370       380       390       400       410       420
                 =========+=========+=========+=========+=========+=========+
Ec-18_003850     RELGGGGSGHGRRGGGNGSGNSSHISKTMPRSTSIASSSSGGHAIVAAAALGGRHTPAAG
MSTRG.48448.1    ------------------------------------------------------------
                                                                             


                        430       440       450       460       470       480
                 =========+=========+=========+=========+=========+=========+
Ec-18_003850     SKPALACELGGTRAANVEEEGGADRGGNELEAEKEALLDYVQDLLGRIKGLEAACASLER
MSTRG.48448.1    ------------------------------------------------------------
                                                                             


                        490       500       510       520       530       540
                 =========+=========+=========+=========+=========+=========+
Ec-18_003850     ERGDSSINLEKTRKDLQQKEKAIAQTQSQIEDLQKTEKVERVEKEEMGRLYNELLKQLKT
MSTRG.48448.1    ---------------------------------------------------MALQNTLSG
                                                                      #######


                        550       560       570       580       590       600
                 =========+=========+=========+=========+=========+=========+
Ec-18_003850     AEEEKEKLQSKAKQADTRKSSISQEANSLREQLDQCLQRLTDEQNCTAESSRQAEEERAD
MSTRG.48448.1    ANSRLAKLDSLEIEVAS---------------------RAAEIQDC----RRKAQ----E
                 ##########                                                  


                        610       620       630       640       650       660
                 =========+=========+=========+=========+=========+=========+
Ec-18_003850     LKNALSSANSRLAKMDALEADTKAQAEELKELREERRERDASLAEGQSRTQHLAGALREL
MSTRG.48448.1    QESALSSAISRLAKLDALEADVKARTAELKKLQREKQESDANLAKERSRTQHLVGAFRDM
                    #########################################################


                        670       680       690       700       710       720
                 =========+=========+=========+=========+=========+=========+
Ec-18_003850     ELESAALNSREREAVSRAEGLEQELHHLESKVSELSLRCEELQKHVVVLKSGEEVQGSRR
MSTRG.48448.1    EDESAILSSRERDALSQAEDVKQELEERGSKVSELSLVCEELQKRIVRLQSGAEMQASRQ
                 ############################################################


                        730       740       750       760       770       780
                 =========+=========+=========+=========+=========+=========+
Ec-18_003850     KAAEAFALAERQDQLLEDTSARLGHLKAESKLLQEELAKTRKSSETAKNILEPVLQGARS
MSTRG.48448.1    QVAEAFALAERQDELLGDASVRLEHLQSEARHLGEELARNRESFEVAKSILVPVLMGARC
                 ############################################################


                        790       800       810       820       830       840
                 =========+=========+=========+=========+=========+=========+
Ec-18_003850     QLGLESGGSCDRHPTTMAAATRKQRRQQAHSFNSECGSQEDFEVENGAGQRQARLDEEGE
MSTRG.48448.1    QLGLRSGGHAHGPTTRMPAAN------QSNSRESGGQPQDDFEMISSDADLRSGDIEEGD
                 ####################       ################                 


                        850       860       870       880       890       900
                 =========+=========+=========+=========+=========+=========+
Ec-18_003850     DE--VELEDLAREVADVLLHVLVGTEVGHG--MGENKEP---------------------
MSTRG.48448.1    EEELVEMEDLAREAAELLLHVLASVSPGISNHVGEATDRPLHRSELSASLPRCVGQGGKS
                     ########################                                


                        910       920       930       940
                 =========+=========+=========+=========+======
Ec-18_003850     --------------------EGRNGRTRWT--------ATRFQDVS
MSTRG.48448.1    RDVFSQHHCRWSMDTDEAGGEGDNGEAQTSSFCGRKAAAVNFDNST
```

```
Parameters used
Minimum Number Of Sequences For A Conserved Position: 2
Minimum Number Of Sequences For A Flanking Position: 2
Maximum Number Of Contiguous Nonconserved Positions: 8
Minimum Length Of A Block: 10
Allowed Gap Positions: None
Use Similarity Matrices: Yes
```

```
Flank positions of the 4 selected block(s)
Flanks: [534  550]  [604  800]  [808  823]  [845  868]  

New number of positions in Ec-18_003850.aa.fasta.trans.aa_ali.fasta-gb:  254  (26% of the original 946 positions)
```
